# Lower versus higher oxygenation targets in ICU patients with haematological malignancy — insights from the HOT-ICU trial

**DOI:** 10.1016/j.bjao.2022.100090

**Published:** 2022-09-23

**Authors:** Thomas L. Klitgaard, Olav L. Schjørring, Marianne T. Severinsen, Anders Perner, Bodil S. Rasmussen

**Affiliations:** 1 Department of Anaesthesia and Intensive Care, Aalborg University Hospital, Aalborg, Denmark; 2 Department of Clinical Medicine, Aalborg University, Aalborg, Denmark; 3 Department of Haematology, Clinical Research Centre, Aalborg University Hospital, Aalborg, Denmark; 4 Department of Intensive Care, Copenhagen University Hospital – Rigshospitalet, Copenhagen, Denmark; 5 Department of Clinical Medicine, University of Copenhagen, Copenhagen, Denmark

**Keywords:** Critical care, Haematologic neoplasms, Hyperoxia, Hypoxia, Oxygen inhalation therapy

## Abstract

**Background:**

Patients admitted to an intensive care unit (ICU) with active haematological malignancy and hypoxaemic respiratory failure have a high mortality. Oxygen supplementation is essential, but limited information exists on the optimum oxygenation targets in these patients.

**Methods:**

This subgroup analysis was specified before completion of the Handling Oxygenation Targets in the ICU (HOT-ICU) trial. The trial investigated the effects of a lower (8 kPa) *vs* a higher (12 kPa) arterial oxygenation target and was stratified for active haematological malignancy, chronic obstructive pulmonary disease, and site. We here report the primary outcome (90-day mortality) and selected secondary outcomes in the subgroup of patients with active haematological malignancy.

**Results:**

The HOT-ICU trial included 168 patients with active haematological malignancy; 82 were randomly allocated to an arterial oxygenation target of 8 kPa, and 86 to 12 kPa. At 90 days, 53/81 patients (65%) in the lower-oxygenation group and 47/86 patients (55%) in the higher-oxygenation group had died: adjusted relative risk 1.22 (95% confidence interval 0.95–1.56); at 1 year, the numbers were 58/81 (72%) *vs* 56/86 (65%): adjusted relative risk 1.11 (95% confidence interval 0.90–1.36). No statistically significant differences were found for any secondary outcomes.

**Conclusion:**

In ICU patients with active haematological malignancies and hypoxaemic respiratory failure, we found a high mortality at 90 days and 1 year. Our results did not preclude clinically relevant benefits or harms of a lower oxygenation target in patients with active haematological malignancy. A randomised trial may, therefore, be worthwhile for these patients.

**Clinical trial registration:**

NCT03174002.

Patients with active haematological malignancy are often immunocompromised, either because of the underlying disease or antineoplastic therapy. Despite treatment advances, this may lead to life-threatening complications, with acute hypoxaemic respiratory failure being a common cause of admission to an intensive care unit (ICU).^1^, ^2^, ^3^, ^4^ ICU admission in turn carries a notoriously high mortality risk,^1^^,^^2^^,^^4^, ^5^, ^6^ comparable to septic shock^7^ and acute respiratory distress syndrome (ARDS),^8^ and higher than in non-haematological patients at both short- and long-term.^2^^,^^9^

The primary supportive treatment for hypoxaemic respiratory failure is supplemental oxygen. Currently, the recommended level of oxygenation in acutely ill adults is targeting an arterial oxygen saturation (SaO_2_) of 92–98%.^10^^,^^11^ However, no specific recommendations exist for patients admitted to the ICU or patients with active haematological malignancy.

The results from the ‘Handling Oxygenation Targets in the ICU’ (HOT-ICU) trial have previously been published; with inclusion of 2928 patients, the trial is the largest to date investigating the benefits and harms of a lower *vs* a higher oxygenation target in ICU patients.^12^^,^^13^ Despite the trial reporting an overall neutral result for both short- and long-term outcomes, important subgroup differences may still be present.^14^, ^15^ Analysis of the effects of targeted oxygen therapy in the stratified subgroup of patients with active haematological malignancy was specified before randomisation of the last patient in the HOT-ICU trial.^16^, ^17^

In addition to the HOT-ICU trial, six additional large-scale randomised clinical trials (RCTs) have investigated the effects of targeted oxygenation in adult patients admitted to the ICU, albeit none have reported data on patients with active haematological malignancies.^18^, ^19^, ^20^, ^21^, ^22^, ^23^ In the HOT-ICU trial, the hypothesis was that a lower oxygenation target would reduce 90-day all-cause mortality as compared with a higher target. We conducted this subgroup analysis to investigate the benefits and harms of a lower *vs* a higher oxygenation target in patients with active haematological malignancy randomised in the HOT-ICU trial.

## Methods

### Trial design

This subgroup analysis was defined before randomisation of the last patient in the HOT-ICU trial and comprised all patients randomised and having active haematological malignancy at baseline.^16^, ^17^ The HOT-ICU trial was a pragmatic, investigator-initiated, multicentre, parallel-group, RCT investigating the benefits and harms of a lower *vs* a higher oxygenation target in patients acutely admitted to the ICU with moderate-to-severe hypoxaemic respiratory failure. This report was prepared in agreement with the Consolidated Standards of Reporting Trials (CONSORT) and the CONSORT checklist is available in the Supplementary material.^24^

The HOT-ICU trial was approved by the Danish Medicines Agency (AAUH-ICU-01, EudraCT number 2017-000632-34); the Committee on Health Research Ethics in the North Denmark Region (N-20170015); the Danish Data Protection Agency (2008-58-0028), and all required authorities in the participating countries; and registered prospectively at ClinicalTrials.gov (NCT03174002). Patient consent was obtained according to national regulations. The protocol, statistical analysis plan, and analysis of the short- and long-term outcomes in the entire cohort have been published.^12^, ^13^, ^16^, ^17^

### Patients

For this sub-study we included patients ≥18 year with active haematological malignancy, acutely admitted to an ICU, receiving oxygen supplementation in a closed system (invasive mechanical ventilation, noninvasive mechanical ventilation, or mask/helmet continuous positive airway pressure) at a fraction of inspired oxygen (FiO_2_) of ≥0.50 or receiving ≥10 L of oxygen min^-^^1^ in an open oxygen supplementation system, with an expected requirement for oxygen supplementation in the ICU for ≥24 h, and having a functioning arterial cannula for arterial blood gas (ABG) sampling. Patients were screened for inclusion within 12 hours of ICU admission.

Active haematological malignancy was defined as any intervention against a predefined list of malignancies including (but not limited to) leukaemia, lymphomas, multiple myeloma, myelodysplastic syndrome, or myeloproliferative neoplasms within 6 months before randomisation, defined from the WHO 2017 classification.^25^ Additional details on definitions of active haematological malignancies, plus inclusion and exclusion criteria, are available in the Supplementary material.

### Randomisation and intervention

Randomisation was performed via a computer-generated allocation sequence with permuted blocks of varying sizes and stratified according to active haematological malignancy (yes/no), chronic obstructive pulmonary disease (COPD) (yes/no), and trial site. Patients were randomised 1:1 to either the lower-oxygenation group (arterial partial pressure of oxygen [PaO_2_] of 8 kPa) or the higher-oxygenation group (PaO_2_ of 12 kPa) and were to adhere to the allocated target during ICU admission for up to 90 days, including any ICU readmissions. Additional details on trial interventions are available elsewhere.^16^ We registered the highest and lowest PaO_2_ in predefined 12-h intervals with concomitant values of FiO_2_ and SaO_2_.

### Outcomes

The primary outcome was all-cause mortality at 90 days post-randomisation. We also report the following secondary outcomes: ‘days alive without life support’ (respiratory support, circulatory support, or renal replacement therapy [RRT]), ‘days alive and out of hospital’, and ‘proportion of patients with one or more serious adverse event (SAE) in the ICU’ (new episodes of shock, cardiac ischaemia, intestinal ischaemia, or cerebral ischaemia), all within 90 days from randomisation; and 1-year all-cause mortality. Additional details on outcome definitions are available in the Supplementary material.

### Statistical analysis

All analyses were performed according to the intention-to-treat principle.^26^ All-cause mortality at 90 days and 1 year, and the proportion of patients with one or more SAE in the ICU were evaluated using a generalised linear model adjusted for COPD with a binomial error distribution and a log-link to produce a relative risk (RR), and an identity link to produce a risk difference. Days alive without life support and days alive and out of hospital were evaluated using the Wilcoxon rank-sum test because of non-normality. Because of non-convergence in the statistical models, no adjustment for trial site was performed in any of these analyses. Mortality analyses were supplemented by Kaplan–Meier plots, Cox proportional hazard models adjusted for COPD and trial site, and logistic regression models with adjustment for COPD, age, and sequential organ failure assessment (SOFA) score. Categorical variables are presented as numbers and percentages, and continuous variables as means and standard deviations or medians and inter-quartile ranges (IQR), as appropriate. No adjustment for multiplicity was performed, and all outcomes are presented with 95% confidence intervals (CIs). All analyses were conducted using STATA statistical software, release 17 (Stata Nordic, Stockholm, Sweden).

## Results

### Patient characteristics

**Fig 1:**
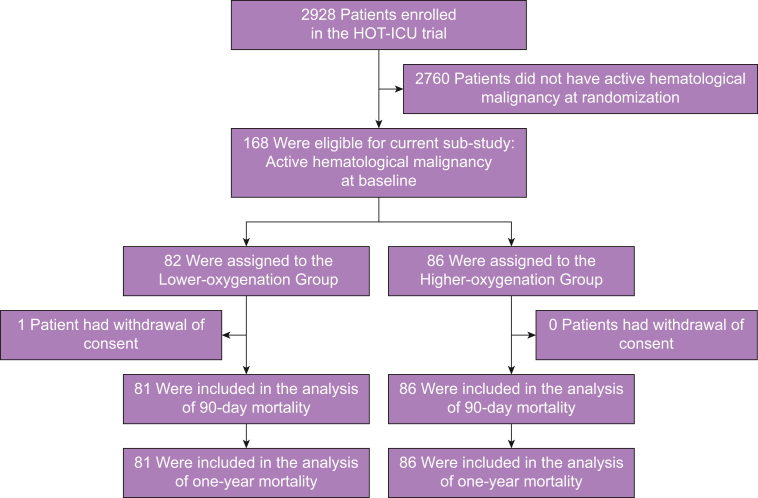
Patient flow. HOT-ICU, Handling Oxygenation Targets in the Intensive Care Unit.

Inclusion in the HOT-ICU trial was conducted from 20 June 2017 to 3 August 2020. In total, 2928 patients were enrolled. Of these, 168 patients had active haematological malignancy at baseline and were thus eligible for the current study; 82 patients were randomly allocated to a target PaO_2_ of 8 kPa and 86 patients to a target PaO_2_ of 12 kPa. One patient in the lower-oxygenation group withdrew consent (Fig. 1). Study groups had similar baseline characteristics (Table 1). Patients included in this sub-study presented with smaller proportions of chronic co-morbidities (e.g. COPD, ischaemic heart disease, or heart failure), but equivalent proportions of acute illnesses (e.g. pneumonia, stroke, or intestinal ischaemia) compared with the remaining HOT-ICU cohort. Patients included in this study displayed similar degrees of hypoxaemic respiratory failure (moderate-to-severe), but were more often on open oxygen supplementation systems than those excluded. Baseline data on patients with and without haematological malignancy are presented in Supplementary Table S1.

**Table 1:** Baseline characteristics of patients with active haematological malignancy.

Variable	Lower-oxygenation group (*N*=82)	Higher-oxygenation group (*N*=86)
Age, median (IQR)	68 (59–73)	68 (64–73)
Male sex, *n* (%)	54 (66)	64 (74)
Median interval between hospital admission and randomisation, days (IQR)	7 (2–16)	4 (1–13)
Median time from ICU admission to randomisation, h (IQR)	4 (2–7)	3 (2–6)
Co-existing illness, *n* (%)		
Ischaemic heart disease	4 (5)	9 (11)
Chronic heart failure	3 (4)	8 (9)
Active metastatic cancer	0 (0)	1 (1)
Long-term dialysis	1 (1)	1 (1)
COPD	8 (10)	8 (9)
Type of admission, *n* (%)		
Medical	78 (95)	80 (93)
Elective surgery	1 (1)	0 (0)
Emergency surgery	3 (4)	6 (7)
Acute illness, *n* (%)		
Pneumonia	49 (60)	54 (63)
Haemorrhagic or ischaemic stroke	1 (1)	1 (1)
Intestinal ischaemia	2 (2)	3 (4)
Cardiac arrest	9 (11)	6 (7)
ARDS	14 (17)	15 (17)
Invasive mechanical ventilation		
Patients, *n* (%)	38 (46)	43 (50)
Median tidal volume, ml (IQR)	504 (430–557)	539 (457–670)
Median end-expiratory pressure, cm H_2_O (IQR)	9 (8–10)	8 (5–10)
Median peak pressure, cm H_2_O (IQR)	24 (22–29)	22 (20–30)
Non-invasive ventilation or CPAP		
Patients, *n* (%)	12 (15)	4 (7)
Median end-expiratory pressure, cm H_2_O (IQR)	8 (6–10)	8 (6–10)
Open system, *n* (%)	32 (39)	39 (45)
Median PaO_2_, kPa (IQR)	10.4 (9.0–12.8)	10.4 (8.6–12.4)
Median SaO_2_, % (IQR)^a^	95 (93–97)	95 (92–96)
Median FiO_2_, fraction (IQR)^b^	0.72 (0.59–1.00)	0.70 (0.59–1.00)
Median PaO_2_:FiO_2_ ratio, kPa (IQR)	15.9 (11.5–20.9)	15.4 (11.4–20.8)
Median lactate concentration, mmol L^-^^1^ (IQR)	1.7 (1.1–3.9)	1.8 (1.2–2.9)
Median lowest mean arterial pressure, mm Hg (IQR)^c^	57 (49–72)	58 (50–73)
Use of vasopressors, *n* (%)	46 (56)	39 (45)
Median highest dose of norepinephrine, μg kg^-^^1^ min^-^^1^ (IQR)	0.29 (0.12–0.60)	0.28 (0.12–0.50)
Median SOFA score (IQR)^d^	9 (8–12)	8 (5–11)

ARDS, acute respiratory distress syndrome; COPD, chronic obstructive pulmonary disease; CPAP, continuous positive airway pressure; FiO_2_, fraction of inspired oxygen; ICU, intensive care unit; IQR, inter-quartile range; PaO_2_, arterial partial pressure of oxygen; SaO_2_, arterial oxygen saturation; SOFA score, sequential organ failure assessment score.

a Data for SaO_2_ were missing for four patients in the lower-oxygenation group and five patients in the higher-oxygenation group because this parameter was not available in blood gas analyses at one site.

b FiO_2_ in open systems was estimated using standardised conversion tables.

c Lowest median value of the mean arterial pressure recorded during the 24 hours before randomisation.

d SOFA scores range from 0 to 24, with higher scores indicating more severe organ failure. Data were missing for one patient in the lower-oxygenation group and two patients in the higher-oxygenation group.

### Oxygenation variables and ICU treatment

**Fig 2:**
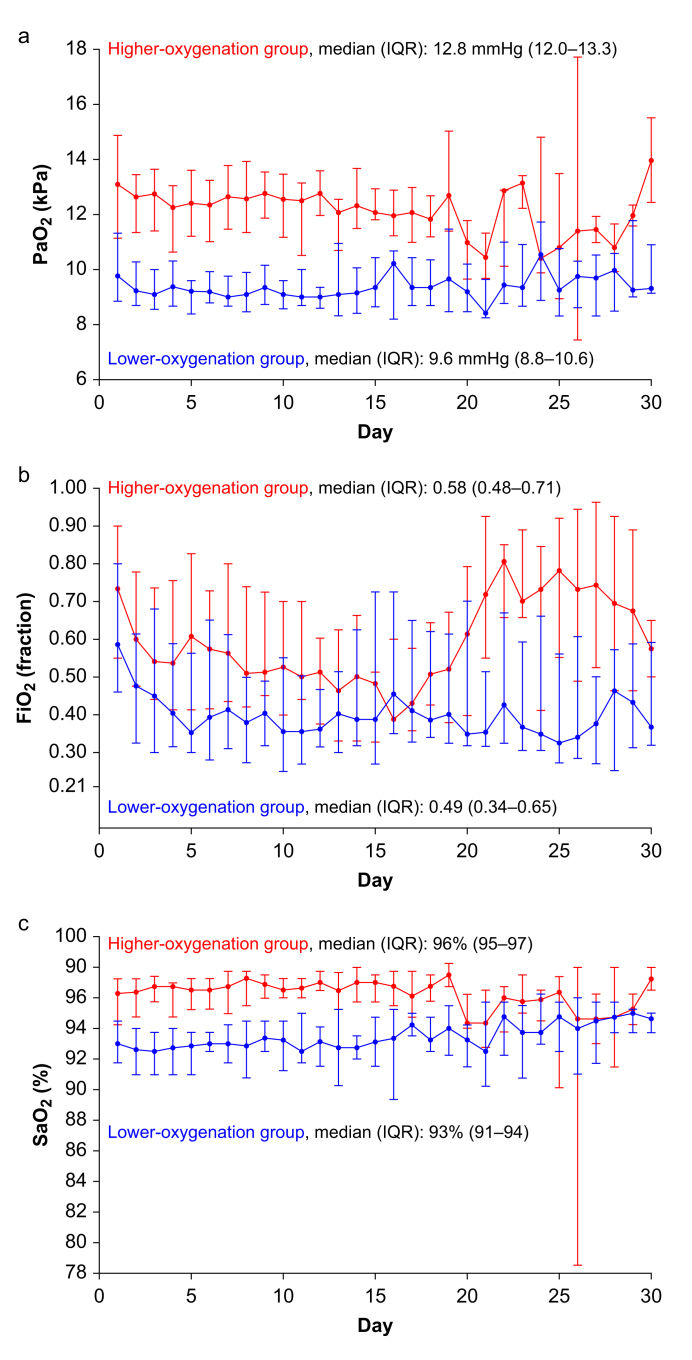
Values of the daily patient-means of PaO_2_, FiO_2_, and SaO_2_ stratified by treatment allocation. Displayed as median values of (a) arterial partial pressure of oxygen (PaO_2_), (b) fraction of inspired oxygen (FiO_2_), and (c) arterial oxygen saturation (SaO_2_) until 30 days after randomisation. Bars represent inter-quartile ranges (IQR). Daily patient-means are calculated from the 12-h highest and lowest PaO_2_ with concomitant FiO_2_ and SaO_2_. Median values for the entire 90-day period are presented. Data for arterial oxygen saturation (SaO_2_) were not available for four patients in the lower-oxygenation group and five patients in the higher-oxygenation group because this parameter was not available in blood gas analyses at one site. Details on the number of patients providing data for each parameter are provided in Supplementary Table S2, and additional data on oxygenation variables are presented in Supplementary Figures S1–S3.

Daily median patient-means of PaO_2_, SaO_2_, and FiO_2_ for the first 30 days are presented in Figure 2. By Day 30, <10% of patients contributed data on oxygenation variables because of death or ICU discharge. Oxygenation data for the entire intervention period are presented in Supplementary Figures S1–S3 and Supplementary Table S2. We found a clear separation in all oxygenation variables for the entire 90-day intervention period: median PaO_2_ in the lower-oxygenation group was 9.6 kPa (IQR 8.8–10.6 kPa) and 12.8 kPa (IQR 12.0–13.3 kPa) in the higher-oxygenation group; median FiO_2_ in the lower-oxygenation group was 0.49 (IQR 0.34–0.65) and 0.58 (IQR 0.48–0.71) in the higher-oxygenation group; median SaO_2_ in the lower-oxygenation group was 93% (IQR 91–94%) and 96% (IQR 95–97%) in the higher-oxygenation group. An average of 7 (±2) ABG analyses were conducted daily per patient in both groups. We found no between-group differences in the use of invasive mechanical ventilation including ventilator settings, use of prone positioning, inhaled vasodilators, extracorporeal membrane oxygenation, red blood cell (RBC) transfusions, vasopressors, or inotropes, or RRT. However, patients with haematological malignancy more often received RRT (33% *vs* 20%) and were more often transfused with RBCs (67% *vs* 31%) than those without haematological malignancy, although cumulated transfused volumes were similar. Details of ICU treatment are available in the Supplementary Tables S3 and S4.

### Outcomes

**Fig 3:**
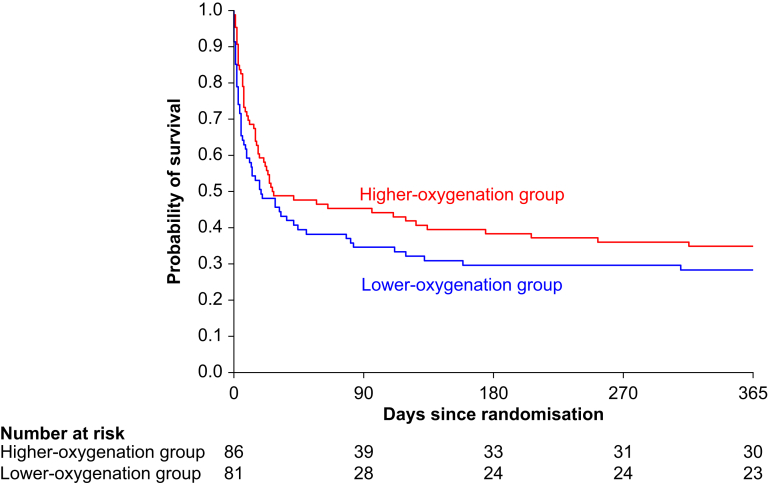
Kaplan–Meier estimates for survival, administratively censored at 365 days. Hazard ratios from Cox proportional-hazards models adjusted for the presence or absence of chronic obstructive pulmonary disease, and trial site: 90-day all-cause mortality: 1.37 (95% CI 0.93–2.03, *P*=0.11); 1-year all-cause mortality: 1.48 (95% CI 0.98–2.26, *P*=0.06).

At Day 90 post-randomisation, 53 of 81 patients (65%) in the lower-oxygenation group and 47 of 86 patients (55%) in the higher-oxygenation group had died: adjusted RR 1.22 (95% CI 0.95–1.56), *P*=0.12. One year after randomisation, 58 of 81 patients (72%) in the lower-oxygenation group and 56 of 86 patients (65%) in the higher-oxygenation group had died: adjusted RR 1.11 (95% CI 0.90–1.36), *P*=0.33 (Table 2). Kaplan–Meier survival plots are presented in Figure 3. When evaluated in the entire HOT-ICU intention-to-treat trial population, no significant interaction between treatment allocation and the presence or absence of active haematological malignancy was found for either 90-day (*P*=0.16) or 1-year all-cause mortality (*P*=0.34). No statistically significant differences were found for any of the other secondary outcomes (Table 2).

**Table 2:** Outcomes.

Variable	Lower-oxygenation group	Higher-oxygenation group	Relative risk (95% CI)	Risk difference (95% CI)	Odds ratio (95% CI)	*P*
Primary outcome						
90-day all-cause mortality, *n*/*n*-total (%)^a^	53/81 (65)	47/86 (55)	1.22 (0.95–1.56)	11.1 (-3.6– 25.9)		0.12
Adjusted for baseline variables^b^					1.27 (0.65–2.49)	0.49
Secondary outcomes						
1-year all-cause mortality, *n*/*n*-total (%)^a^	58/81 (72)	56/86 (65)	1.11 (0.90–1.36)	6.8 (-7.3–20.9)		0.33
Adjusted for baseline variables^b^					1.09 (0.54–2.20)	0.82
Median number of days alive without life-support (IQR)^c^^,^^d^	4 (0–81)	24 (0–84)				0.37
Median number of days alive and out of hospital (IQR)^c^	0 (0–52)	0 (0–59)				0.26
Serious adverse events in the ICU, *n*/*n*-total (%)^c^	34/82 (41)	38/86 (44)	0.97 (0.68–1.37)	−2.7 (−17.6– 12.1)		0.86
New episode of shock	31/82 (38)	36/86 (42)				
New myocardial infarction	1/82 (1)	3/86 (3)				
New ischaemic stroke	1/82 (1)	0/86 (0)				
New intestinal ischaemia	3/82 (4)	1/86 (1)				

*P*-values are presented for the relative risk or odds ratio. Relative risks and risk differences are adjusted for the presence or absence of COPD. COPD, chronic obstructive pulmonary disease; ICU, intensive care unit; SOFA, sequential organ failure assessment.

a Data for mortality was missing for one patient in the lower-oxygenation group. Relative risks and risk differences are adjusted for the presence or absence of COPD.

b Baseline variables were presence or absence of COPD, age, and SOFA score which ranges from 0 to 24, with higher scores indicating more severe organ failure.

c Within 90 days.

d Life support was defined as the use of invasive mechanical ventilation, noninvasive ventilation, continuous positive airway pressure (non-intermittently), vasopressor or inotropic infusion, or any type of renal replacement therapy.

## Discussion

The HOT-ICU trial is the largest trial to date investigating the effects of a lower *vs* a higher oxygenation strategy in patients acutely admitted to the ICU. In this sub-study in patients with active haematological malignancy acutely admitted to the ICU with moderate-to-severe hypoxaemic respiratory failure, targeting a PaO_2_ of 8 kPa did not result in reduced 90-day all-cause mortality compared with targeting 12 kPa. Also, we found no differences in any secondary outcomes, including 1-year mortality. Importantly, we did not find any significant difference in the occurrence of SAEs, with most SAEs being new episodes of shock, and few ischaemic events in either group (Table 2). Point estimates for both short- and long-term mortality, coupled with no apparent increase in the risk of adverse events, were thus suggestive of the potential benefit of a higher oxygenation target. However, CIs for effect estimates did not preclude potentially important clinical harm or benefit of the lower oxygenation strategy, thereby underscoring the need for larger trials to inform clinical practice.

We included 168 patients with active haematological malignancy, equivalent to 6% of the entire cohort, and thus more than the expected case-mix proportion of 2–3%.^27^ The low proportion of patients with active haematological malignancy in the ICU in general could be caused by clinicians' reluctance to admit such patients to the ICU because of the expected high mortality risk or unclear prognosis.^28^ However, survival among haematological patients has increased in recent years, both in general^29^ and in those admitted to the ICU.^9^, ^27^, ^30^ The overall mortality of patients included in the present subgroup analysis was higher than in the remaining HOT-ICU cohort at both 90 days (60% *vs* 42%) and 1 year (68% *vs* 48%). Our reported mortality rates are in line with previous observational studies of patients with haematological malignancies admitted to the ICU,^2^, ^27^ but higher than others.^1^, ^4^, ^5^, ^6^, ^9^ Despite excluding patients for whom active therapy was withdrawn before randomisation, the studied cohort still had a high mortality, thus emphasising the need for further improvement of therapy for this subset of patients.

No baseline information on immunosuppression or malignancies was provided in the first of the larger RCTs on oxygenation targeting in the ICU, the OXYGEN-ICU trial.^18^ In the HYPERS2S trial, 20% of patients were immunosuppressed at baseline (*N* = 87/434), and the trial suggested overall benefit (decreased 28-day mortality) of a lower oxygenation strategy in patients admitted to the ICU with sepsis, although this was not statistically significant.^19^ The ICU-ROX trial investigated a conservative oxygenation strategy *vs* conventional therapy in mechanically ventilated ICU patients, and 10% of patients were immunosuppressed at inclusion (*N* = 100/965); the study demonstrated no difference between the oxygenation strategies overall.^20^ Eleven percent of patients included in the LOCO_2_ trial were immunosuppressed at baseline (*N* = 22/201), and overall the trial suggested benefit of a higher oxygenation strategy in ICU patients with ARDS.^21^ Investigators of the O_2_-ICU trial reported a comparable proportion of patients with haematological malignancy at baseline as in our study (5%, *N* = 20/400); overall, the trial did not demonstrate any difference in mortality when comparing a low-normal to a high-normal oxygenation strategy in ICU patients with systemic inflammation.^22^ In the recently publisehd BOX trial, investigating targeted oxygen therapy in comatose survivors of out-of-hospital cardiac arrest, 1% of patiens had haematological malignancy at baseline (10/789); the trial resulted in no differences in the incidences of death or severe disability or coma between the groups.^23^ Unfortunately, none of the aforementioned RCTs have reported results specifically for immunosuppressed patients or those with haematological malignancies.

Infection with SARS-CoV-2 may also lead to a dysregulated immune response,^31^ yet when comparing the two oxygenation targets in the subset of 110 patients with COVID-19 in the HOT-ICU trial, we found no difference in 90-day mortality.^32^ Because of the pragmatic design of the HOT-ICU trial, we are not able to *post hoc* identify patients with immunosuppression at baseline attributable to other causes (e.g. high-dose steroid treatment or cancer therapy). Also, we acknowledge that one cannot necessarily equate baseline mortality risks or susceptibility to effects of targeted oxygen therapy of immunocompromised patients (as a result of e.g. COVID-19 or immunomodulatory therapy) to those with active haematological malignancy. However, patients presenting with both COVID-19 and haematological malignancy should still be considered for ICU admission as per a recent systematic review and meta-analysis.^33^

Our findings did not support the overall trial hypothesis, that a lower oxygenation target would reduce mortality as compared with a higher target. Hypoxia has been suggested to favour haematological tumour progression, relapse, and resistance to chemotherapy via hypoxia-inducible transcription factor pathways.^34^ Conversely, hyperbaric oxygen therapy has been suggested to inhibit cancer progression, although the evidence supporting this is still limited.^35^, ^36^ However, hyperoxaemia is generally discouraged among ICU patients because of the risks of oxygen toxicity (e.g. cerebral and cardiac vasoconstriction, increased production of reactive oxygen species, and pulmonary absorption atelectasis).^37^ Also, concerns of reduced cancer-free survival in patients undergoing abdominal surgery if subjected to high perioperative FiO_2_ have been raised.^38^ An arterial saturation of 92–98% is generally recommended by the latest guidelines for acutely ill patients in general,^10^, ^11^ and is comparable to the estimate in the HOT-ICU trial for the higher-oxygenation group when considering the entire 90-day intervention period (median SaO_2_ 96%, IQR 95–97%). However, the most recent systematic review on oxygenation strategies in acutely ill patients broadly did not find any evidence for beneficial or harmful effects of higher oxygenation strategies compared with lower, though the certainty of evidence was low to very low.^39^ Albeit none of the included trials investigated the effects of targeted oxygenation therapy in patients with haematological malignancies specifically, nor have reported any separate analyses for such patients.

The strengths and limitations of the HOT-ICU trial are all carried over to this sub-study and are discussed in detail elsewhere.^12^, ^13^ Of additional strength, randomisation in the HOT-ICU trial was stratified for the presence or absence of active haematological malignancy, and baseline characteristics were similar in the two groups (Table 1), thus enabling valid inferences to be drawn from this subset of patients.^40^ Stratification for active haematological malignancy was performed because of an expected higher mortality in this subset of patients compared with the remaining cohort — which was confirmed. Also, patients for whom withdrawal of active therapy was deemed imminent were ineligible, thus emphasising a focus on patients in whom the intervention could have an effect. We recruited patients from 23 sites in six countries, enabling generalisability, and observed excellent separation in oxygenation variables for the entire intervention period. As in the main cohort of the HOT-ICU trial, patients in the lower-oxygenation group presented with a higher-than-intended median PaO_2_ (9.6 *vs* 8 kPa) when considering the entire intervention period.^12^ As for the main cohort, we suspect this deviation to have several causes: clinicians' hesitance with targeting patients' oxygenation below 8 kPa; the fact that we only registered the 12-h highest and lowest PaO_2_ values, thus lending weight to the higher extreme measurements; and that patients may achieve PaO_2_ values >8 kPa despite FiO_2_=0.21. However, a median between-group difference >3 kPa was still achieved, which is greater than in most of the other large-scale ICU RCTs on targeted oxygenation strategies,^18^, ^19^, ^20^, ^22^ and similar to the LOCO_2_ trial.^21^

We had predefined a list of diagnoses qualifying as haematological malignancies (Supplementary material) to inform stratification and the individual haematological diagnoses were not registered.

Also, patients included in this sub-study were treated similarly during ICU stay to the remaining cohort except for higher proportions receiving RBC transfusion or RRT. Although haematological patients may be anaemic (haemoglobin levels were not part of data registration), patients in the current sub-study were transfused with comparable volumes of RBC to those excluded (Supplementary material).

Finally, our study was underpowered to detect the suggested mortality effects. To detect a 90-day all-cause mortality difference as suggested, a total of 656 patients would be needed (alpha 5%; beta 20%).

In conclusion, in patients with active haematological malignancy and acute moderate-to-severe hypoxaemic respiratory failure, a lower oxygenation target as compared with a higher target did not reduce 90-day all-cause mortality. Although defined before trial completion, and stratified for the condition in question, the current study must be regarded as exploratory only. Correspondingly, the findings need to be corroborated in future large-scale clinical trials to inform clinical practice.

## Authors' contributions

Wrote the manuscript: TLK

Read and approved the final manuscript: all authors

Coordinating investigators of the HOT-ICU trial: TLK, OLS

Sponsor of the HOT-ICU trial: BSR

Part of the HOT-ICU trial’s steering committee: TLK, OLS, AP, BSR

## Declarations of interest

The Department of Intensive Care at Rigshospitalet has received funding for other projects from the Novo Nordisk Foundation, Pfizer, Sygeforsikringen ‘danmark’ and Fresenius Kabi, and does contract research for AM-Pharma.

## Funding

The HOT-ICU trial was supported by Innovation Fund Denmark (4108-00011A), Aalborg University Hospital, the Regions of Denmark (EMN-2017-00901, EMN-2019-01055), the Obel Family Foundation (25457), the Danish Society of Anaesthesiology and Intensive Care Medicine, and the Intensive Care Symposium Hindsgavl. No additional funding was provided for this sub-study.

## Data sharing statement

Complete deidentified patient data set collected during the HOT-ICU will be shared beginning two years after April 8, 2021 with no end date. Data will be available to researchers who provide a methodologically sound proposal for the purposes of achieving specified aims. The proposals will be reviewed by the HOT-ICU trial Management Committee. To gain access, the researchers will need to sign a data access agreement and to confirm that data will only be used for the agreed purpose for which access was granted. Request for data must be sent to the primary investigator and HOT-ICU sponsor via email: bodil.steen.rasmussen@rn.dk.

## Code availability

Can be shared upon request.
