# Supplementary material for: Lower versus higher oxygenation targets in ICU patients with haematological malignancy — insights from the HOT-ICU trial
Source: BJA Open. 2022 Sep 23;4:100090. doi: 10.1016/j.bjao.2022.100090 (PMC10430820; doi:10.1016/j.bjao.2022.100090)
Supplement: Supplementary file 1 — Multimedia component 1 [file mmc1.pdf]

Lower versus higher oxygenation targets in ICU patients with haematological malignancy – insights from the HOT-ICU trial

Thomas Lass Klitgaard[1,2,*], Olav Lilleholt Schjørring[1,2], Marianne Tang Severinsen[2,3], Anders Perner[4,5], Bodil Steen Rasmussen[1,2]

[1]Department of Anaesthesia and Intensive Care, Aalborg University Hospital, Aalborg Denmark

[2]Department of Clinical Medicine, Aalborg University, Aalborg, Denmark

[3]Department of Haematology, Clinical Research Centre, Aalborg University Hospital, Aalborg Denmark

[4]Department of Intensive Care, Copenhagen University Hospital – Rigshospitalet, Copenhagen Denmark

[5]Department of Clinical Medicine, University of Copenhagen, Copenhagen, Denmark

*Corresponding author. Email: tlk@rn.dk

Electronic Supplementary Material

Table of content

Inclusion and exclusion criteria for the HOT-ICU trial 2

Definition of active haematological malignancy 3

Outcome definitions 4

Table S1 Baseline characteristics of patients with and without active haematological malignancy 6

Table S2 Number of patients providing data on oxygenation 8

Table S3 Intensive care unit treatment parameters in patients with active haematological malignancy 9

Table S4 Intensive care unit treatment parameters in patients *with* and *without* active haematological malignancy 10

Fig. S1 Daily patient-mean PaO_2 11

Fig. S2 Daily patient-mean FiO_2 12

Fig. S3 Daily patient-mean SaO_2 13

Inclusion and exclusion criteria for the HOT-ICU trial

Inclusion criteria

- Acute admission to the intensive care unit (ICU)
- ≥18 years
- Supplemental oxygen:
    - ≥10 litres of oxygen per minute in an open system including high-flow systems OR
    - FiO_2 ≥0.50 in a closed system (invasive mechanical ventilation, non-invasive [mask or helmet] ventilation, or continuous positive airway pressure [CPAP] systems [mask or helmet])
- Expected ≥24 hours of oxygen supplementation in the ICU
- Functioning arterial line

Exclusion criteria

- Cannot be randomised within 12 hours of ICU admission
- Receives chronic mechanical ventilation (invasive mechanical ventilation, continuous non-invasive ventilation, or continuous mask-CPAP) for any reason
- Use of supplementary oxygen at home
- Previous treatment with bleomycin
- Organ transplant planned or performed during current hospitalisation
- Withdrawal from active therapy or brain death deemed imminent
- Pregnancy
- Poisoning with carbon monoxide, cyanide, or paraquat
- Methemoglobinemia
- Sickle cell disease
- Use of hyperbaric oxygen treatment
- Consent not obtainable according to national regulations
- Previously randomised into the HOT-ICU trial

Definition of active haematological malignancy

Any interventions within the last six months against any of the following:

- Leukaemia: acute lymphoblastic leukaemia (ALL), acute myelogenous leukaemia (AML), chronic lymphocytic leukaemia (CLL), hairy cell leukaemia (HCL), T-cell prolymphocytic leukaemia (T-PLL), B-cell prolymphocytic (B-PLL), large granular lymphocytic leukaemia (LGL)
- Lymphoma: Hodgkin's lymphomas, Non-Hodgkin's lymphoma (e.g. small lymphocytic lymphoma (SLL), lymphoblastic lymphoma, diffuse large B-cell lymphoma (DLBCL), follicular lymphoma (FL), mantle cell lymphoma (MCL), marginal zone lymphoma (MZL), Burkitt's lymphoma (BL), post-transplant lymphoproliferative disorder (PTLD), Waldenström's macroglobulinaemia, NK- or T-cell lymphomas).
- Multiple myeloma/plasma cell myeloma, solitary plasmacytoma
- Myelodysplastic syndromes
- Myeloproliferative neoplasms (MPN) (e.g. chronic myelogenous leukaemia (CML), chronic neutrophilic leukaemia, primary myelofibrosis (PMF), myeloproliferative neoplasm, unclassifiable, Mast cell diseases
- Other (rare) malignant lymphoid and myeloid diseases
- Benign haematological diseases: aplastic anaemia, autoimmune haemolytic anaemia

The list is defined from the WHO 2017 classification:

Swerdlow SH, Campo E, Harris NL, et al. WHO Classification of Tumours of Haematopoietic and Lymphoid Tissues, Revised Fourth Edition WHO Classification of Tumours, IARC WHO Classification of Tumours, No 2. 4th ed. WHO Press; 2017.

Outcome definitions

Primary outcome:

- 90-day mortality: death from any cause within 90 days following the day of randomisation

Secondary outcomes:

- One-year mortality: death from any cause within one year following the day of randomisation
- Proportion of patients with one or more of the following serious adverse events in the ICU after randomisation:
  - *New myocardial ischaemia:* ST-elevation myocardial infarction, non-ST-elevation myocardial infarction, or unstable angina pectoris according to the criteria of the clinical condition in question, and receiving treatment as a consequence of this (reperfusion strategies, or initiation of or increased antithrombotic treatment)
  - *New ischaemic stroke:* cerebral computerised tomography (CT) or magnetic resonance imaging (MRI) scan conducted on this day with signs of new ischaemic stroke. Radiographic signs of old infarctions estimated to have occurred before randomisation are not considered a new ischaemic stroke. Radiographically diagnosed diffuse anoxic brain injury after pre- randomisation cardiac arrest is not considered a new ischaemic stroke
  - *New intestinal ischaemia:* onset of gastric, mesenteric, or colonic ischaemia on this day, verified by exploratory or diagnostic abdominal surgery, endoscopic procedures or by CT or MRI angiography
  - *New episode of shock:* plasma lactate concentration (p-lactate) >2.0 mmol per litre and the use of continuous vasopressor or inotropic treatment on any day in the ICU in participants who did not have shock at baseline (p-lactate ≤2.0 mmol per litre or no use of continuous vasopressor or inotropic treatment) or who were shock-free on any of the previous post-randomisation days. Shock-free on any day is defined as the highest daily p-lactate ≤2.0 mmol per litre and no use of vasopressor or inotropic treatment
- Days alive without the use of respiratory support (invasive mechanical ventilation, non-invasive ventilation, or non-intermittent CPAP), circulatory support (continuous infusion of vasopressor or inotrope) or any form of renal replacement therapy (in patients receiving intermittent renal replacement therapy days between treatments are included as being with the use of renal replacement therapy) in the 90 days after randomisation
- Days alive and out of the hospital in the 90 days after randomisation

Table S1 Baseline characteristics of patients with and without active haematological malignancy

Variable	Patients *with* active haematological malignancy	Patients *without* active haematological malignancy
**n**	168	2742
**Age, median (IQR)**	68 (61–73)	70 (60–77)
**Male sex, n (%)**	118 (70)	1753 (64)
**Median interval between hospital admission and randomisation, days (IQR)**	6 (1–15)	1 (0–4)
**Median time from ICU admission to randomisation, hours (IQR)**	4 (2–7)	4 (2–7)
**Co-existing illness, n (%)**		
Ischaemic heart disease	13 (8)	397 (15)
Chronic heart failure	11 (7)	275 (10)
Active metastatic cancer	1 (1)	125 (5)
Long-term dialysis	2 (1)	45 (2)
COPD	16 (10)	547 (20)
**Type of admission, n (%)**		
Medical	158 (94)	2330 (85)
Elective surgery	1 (1)	38 (1)
Emergency surgery	9 (5)	374 (14)
**Acute illness, n (%)**		
Pneumonia	103 (61)	1571 (57)
Multiple trauma	0 (0)	53 (2)
Haemorrhagic or ischaemic stroke	2 (1)	45 (2)
Traumatic brain injury	0 (0)	24 (1)
Myocardial infarction	6 (4)	177 (7)
Intestinal ischaemia	5 (3)	63 (2)
Cardiac arrest	15 (9)	320 (12)
ARDS	29 (17)	344 (13)
**Invasive ventilation**		
Patients, n (%)	81 (48)	1623 (59)
Median tidal volume, ml (IQR)	516 (453–632)	499 (428–570)
Median end-expiratory pressure, cmH_2O (IQR)	8 (6–10)	9 (7–10)
Median peak pressure, cmH_2O (IQR)	24 (20–29)	25 (21–29)
**Non-invasive ventilation or CPAP**		
Patients, n (%)	16 (10)	359 (13)
Median end-expiratory pressure, cmH_2O (IQR)	8 (6–10)	7 (5–8)
**Open system, n (%)**	71 (42)	760 (28)

**Median PaO_2, kPa (IQR)**	10.4 (9.0–12.6)	10.3 (8.7–12.4)
**Median SaO_2, % (IQR)***	95 (92–97)	94 (91–97)
**Median FiO_2, fraction (IQR)†**	0.70 (0.59–1.00)	0.70 (0.55–0.85)
**Median PaO_2:FiO_2 ratio, kPa (IQR)**	15.8 (11.5–20.8)	15.7 (119–20.7)
**Median lactate concentration, mmol $litre^{-1}$ (IQR)**	1.7 (1.1–3.7)	1.8 (1.1–3.1)
**Median lowest mean arterial pressure, mmHg (IQR)‡**	58 (49–73)	58 (48–68)
**Use of inotropes, n (%)**	1 (1)	69 (3)
**Use of vasopressors, n (%)**	85 (55)	1506 (55)
Median highest dose of epinephrine, µg kg^{-1} min^{-1} (IQR)	0.28 (0.12–0.55)	0.20 (0.10–0.40)
**Median SOFA score (IQR)§**	9 (6–12)	8 (5–10)

ICU denotes intensive care unit, IQR inter-quartile range, COPD chronic obstructive pulmonary disease, ARDS acute respiratory distress syndrome, CPAP continuous positive airway pressure, PaO_2 arterial partial pressure of oxygen, SaO_2 arterial oxygen saturation, FiO_2 fraction of inspired oxygen, SOFA score sequential organ failure assessment score.

*Data for arterial oxygen saturation (SaO_2) were missing for 182 patients in the subgroup of patients without haematological malignancy and nine in the subgroup of patients with active haematological malignancy because this parameter was not available in blood gas analyses at one site.

†FiO_2 in open systems was estimated using standardised conversion tables.

‡Lowest median value of the mean arterial pressure was recorded during the 24 hours before randomisation.

§SOFA scores range from 0 to 24, with higher scores indicating more severe organ failure. Data were missing for 92 patients in the subgroup of patients without haematological malignancy and for three in the subgroup of patients with active haematological malignancy.

Table S2 Number of patients providing data on oxygenation

	PaO_2		FiO_2		SaO_2	
**Day**	**Lower-oxygenation Group**	**Higher-oxygenation Group**	**Lower-oxygenation Group**	**Higher-oxygenation Group**	**Lower-oxygenation Group**	**Higher-oxygenation Group**
**1**	82	84	82	84	78	79
**5**	46	58	46	57	42	55
**10**	27	31	27	31	25	30
**15**	15	17	15	17	14	17
**20**	11	8	11	8	11	8
**25**	11	4	11	4	11	4
**30**	10	2	10	2	10	2
**35**	6	3	6	3	6	3
**40**	2	3	2	3	2	3
**45**	2	2	2	2	2	2
**50**	3	2	3	2	3	2
**55**	2	1	2	1	2	1
**60**	2	0	2	0	2	0
**65**	1	1	1	1	1	1
**70**	1	0	1	0	1	0
**75**	1	1	1	1	1	1
**80**	1	0	1	0	1	0
**85**	1	0	1	0	1	0
**90**	0	0	0	0	0	0

PaO_2 denotes partial pressure of arterial oxygen, FiO_2 fraction of inspired oxygen, SaO_2 arterial oxygen saturation. The number of patients with active haematological malignancy and data on oxygenation parameters stratified by treatment allocation.

Data on SaO_2 were missing for four patients in the lower-oxygenation group and five patients in the higher-oxygenation group because this parameter was not available in blood gas analyses at one site.

Table S3 Intensive care unit treatment parameters in patients with active haematological malignancy

Variable	Lower-oxygenation Group	Higher-oxygenation Group
**n**	82	86
**Mean number of daily ABGs (SD)**	7 (2)	7 (2)
**Mechanical ventilation, n (%)**		
Any use of invasive mechanical ventilation	62 (76)	67 (78)
Any use of non-invasive ventilation or CPAP	18 (22)	20 (23)
**Invasive mechanical ventilation***		
Median tidal volume per kg predicted body weight, millilitres kg^{-1} (IQR)[†]	7.5 (6.6–8.7)	7.5 (6.8–8.3)
Median PEEP, cmH_2O (IQR)	9 (8–11)	10 (8–12)
Median peak pressure, cmH_2O (IQR)	21 (18–27)	24 (19–27)
**Any use of prone positioning, n (%)**	5 (6)	8 (9)
**Any use of inhaled vasodilators, n (%)[‡]**	3 (4)	2 (2)
**Any use of extracorporeal membrane oxygenation, n (%)**	0 (0)	2 (2)
**Any use of vasopressors or inotropes, n (%)[§]**	67 (82)	70 (81)
**Any use of renal replacement therapy, n (%)[¶]**	24 (29)	32 (37)
**Any use of blood transfusions, n (%)**	57 (70)	56 (65)
Median number of blood transfusions (IQR)[‖]	3 (2–6)	3 (1–5)
Median transfused volume, ml (IQR)[‖]	735 (490–1500)	735 (300–1360)
**Non-invasive ventilation or CPAP**		
Median EPAP or CPAP, cmH_2O (IQR)	7 (6–10)	8 (6–10)

ABG denotes arterial blood gas sample, SD standard deviation, PEEP positive end-expiratory pressure, CPAP continuous positive airway pressure, EPAP end-expiratory pressure.

*Parameters were registered once daily at 08:00 hours.

[†]Predicted body weight: 50 kg + 0.91 kg/cm x (height – 152.4 cm) for males and 45.5 kg + 0.91 kg/cm x (height – 152.4 cm) for females.

[‡]Any use of inhaled nitric oxide or epoprostenol.

[§]Continuous infusion of dopamine, norepinephrine, epinephrine, phenylephrine, vasopressin, dobutamine, milrinone, or levosimendan.

[¶]Any continuous or intermittent use of renal replacement therapy.

[‖]In patients receiving red blood cell transfusion.

Table S4 Intensive care unit treatment parameters in patients *with* and *without* active haematological malignancy

Variable	Patients *with* active haematological malignancy	Patients *without* active haematological malignancy
**n**	168	2742
**Mean number of daily ABGs (SD)**	7 (2)	6 (2)
**Mechanical ventilation, n (%)**		
Any use of invasive mechanical ventilation	129 (80)	2083 (76)
Any use of non-invasive ventilation or CPAP	38 (27)	611 (22)
**Invasive mechanical ventilation***		
Median tidal volume per kg predicted body weight, millilitres kg^{-1} (IQR)†	7.5 (6.6–8.5)	7.3 (6.4–8.2)
Median PEEP, cmH_2O (IQR)	9 (8–12)	9 (8–10)
Median peak pressure, cmH_2O (IQR)	23 (18–27)	22 (19–26)
**Any use of prone positioning, n (%)**	13 (8)	154 (7)
**Any use of inhaled vasodilators, n (%)‡**	5 (3.0)	119 (4)
**Any use of extracorporeal membrane oxygenation, n (%)**	2 (1)	24 (1)
**Any use of vasopressors or inotropes, n (%)§**	137 (82)	2222 (81)
**Any use of renal replacement therapy, n (%)¶**	56 (33)	547 (20)
**Any use of blood transfusions, n (%)**	113 (67)	841 (31)
Median number of blood transfusions (IQR)‖	3 (2–6)	3 (1–5)
Median transfused volume, ml (IQR)‖	735 (490–1500)	735 (300–1500)
**Non-invasive ventilation or CPAP**		
Median EPAP or CPAP, cmH_2O (IQR)	8 (6–10)	8 (6–9)

ABG denotes arterial blood gas sample, SD standard deviation, PEEP positive end-expiratory pressure, CPAP continuous positive airway pressure, EPAP end-expiratory pressure.

*Parameters were registered once daily at 08:00 hours.

†Predicted body weight: 50 kg + 0.91 kg/cm x (height – 152.4 cm) for males and 45.5 kg + 0.91 kg/cm x (height – 152.4 cm) for females.

‡Any use of inhaled nitric oxide or epoprostenol.

§Continuous infusion of dopamine, norepinephrine, epinephrine, phenylephrine, vasopressin, dobutamine, milrinone, or levosimendan.

¶Any continuous or intermittent use of renal replacement therapy.

‖In patients receiving red blood cell transfusion.

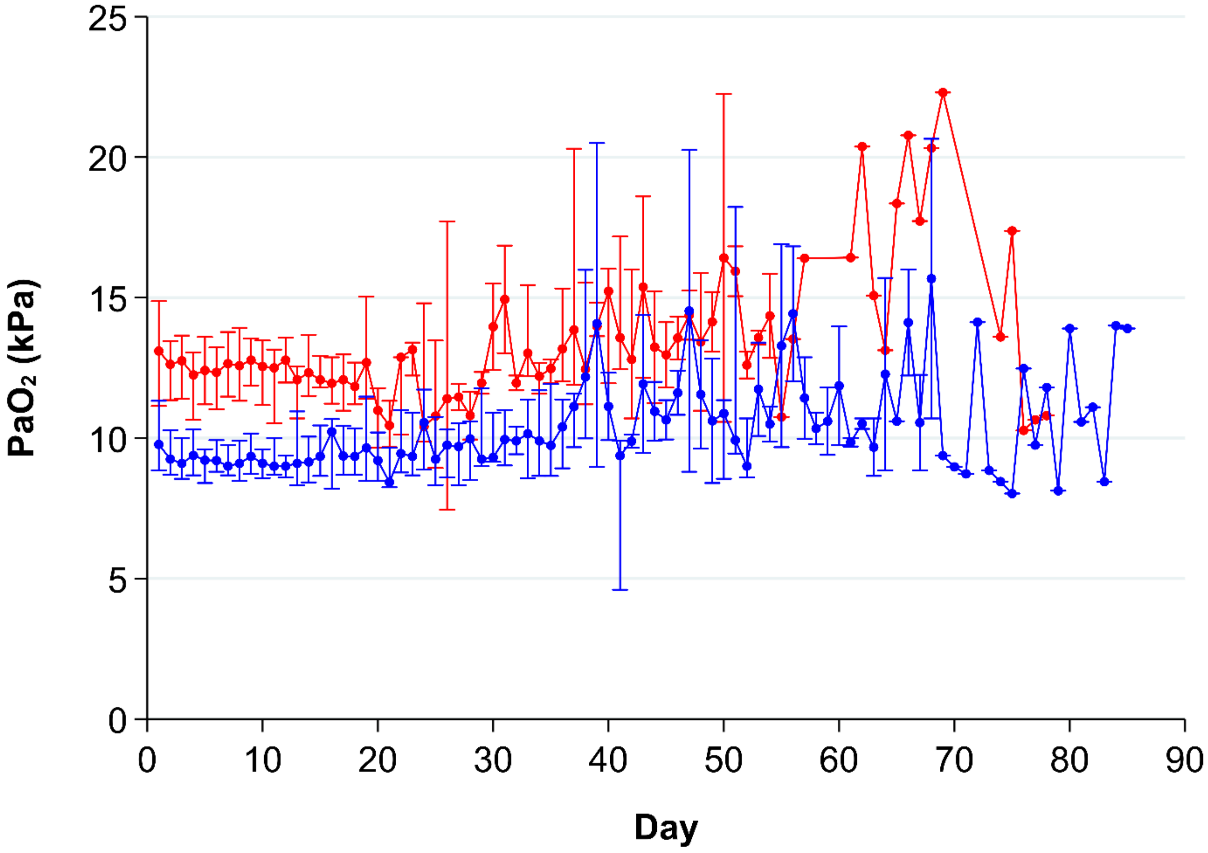

Fig. S1 Daily patient-mean PaO_2

Median values of daily patient-means of partial pressure of arterial oxygen (PaO_2) stratified according to oxygenation target allocation for the 90-day intervention period in patients with active haematological malignancy.

Daily patient-means were calculated from the registered 12-hour highest and lowest values. Bars represent interquartile ranges (IQR).

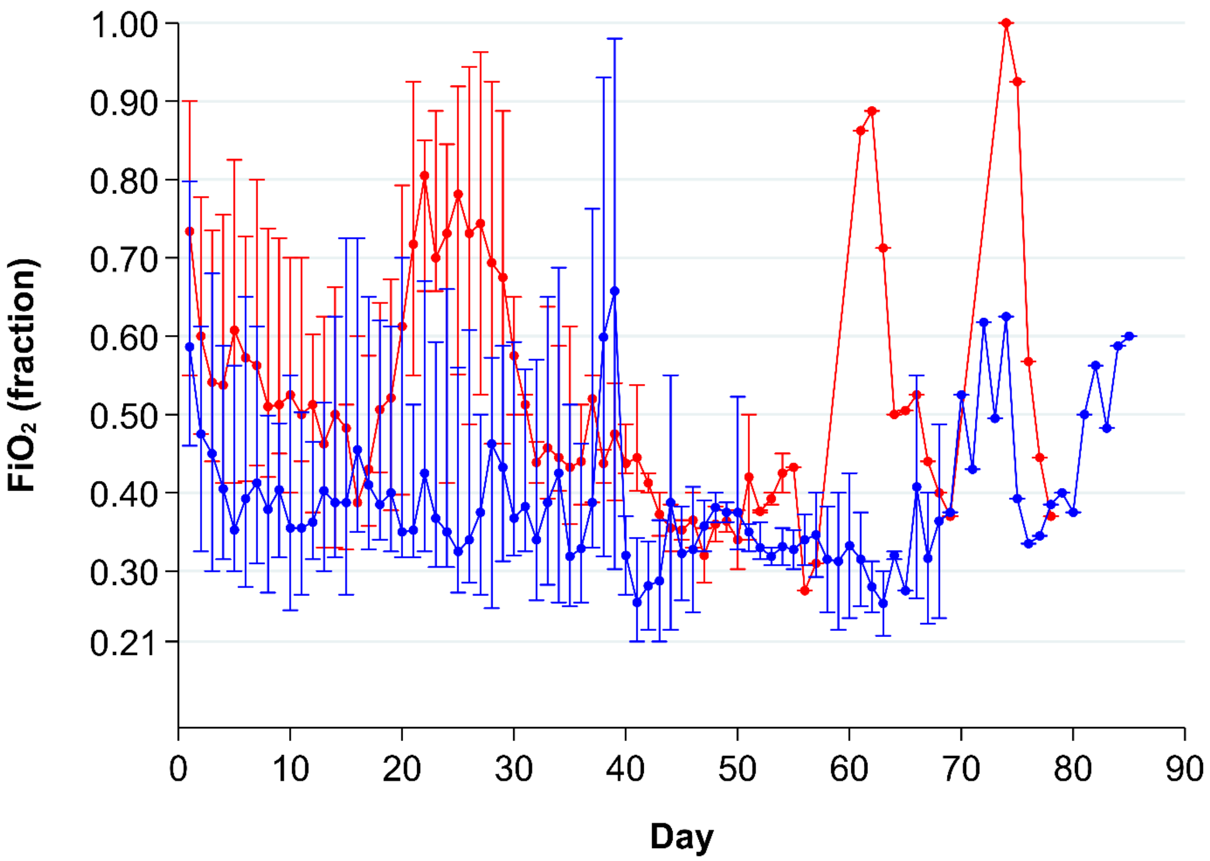

Fig. S2 Daily patient-mean FiO_2

Median values of daily patient-means of fractions of inspired oxygen (FiO_2) stratified according to oxygenation target allocation for the 90-day intervention period in patients with active haematological malignancy.

Daily patient-means were calculated from the registered values corresponding to the 12-hour highest and lowest partial pressure of arterial oxygen. Bars represent interquartile ranges (IQR).

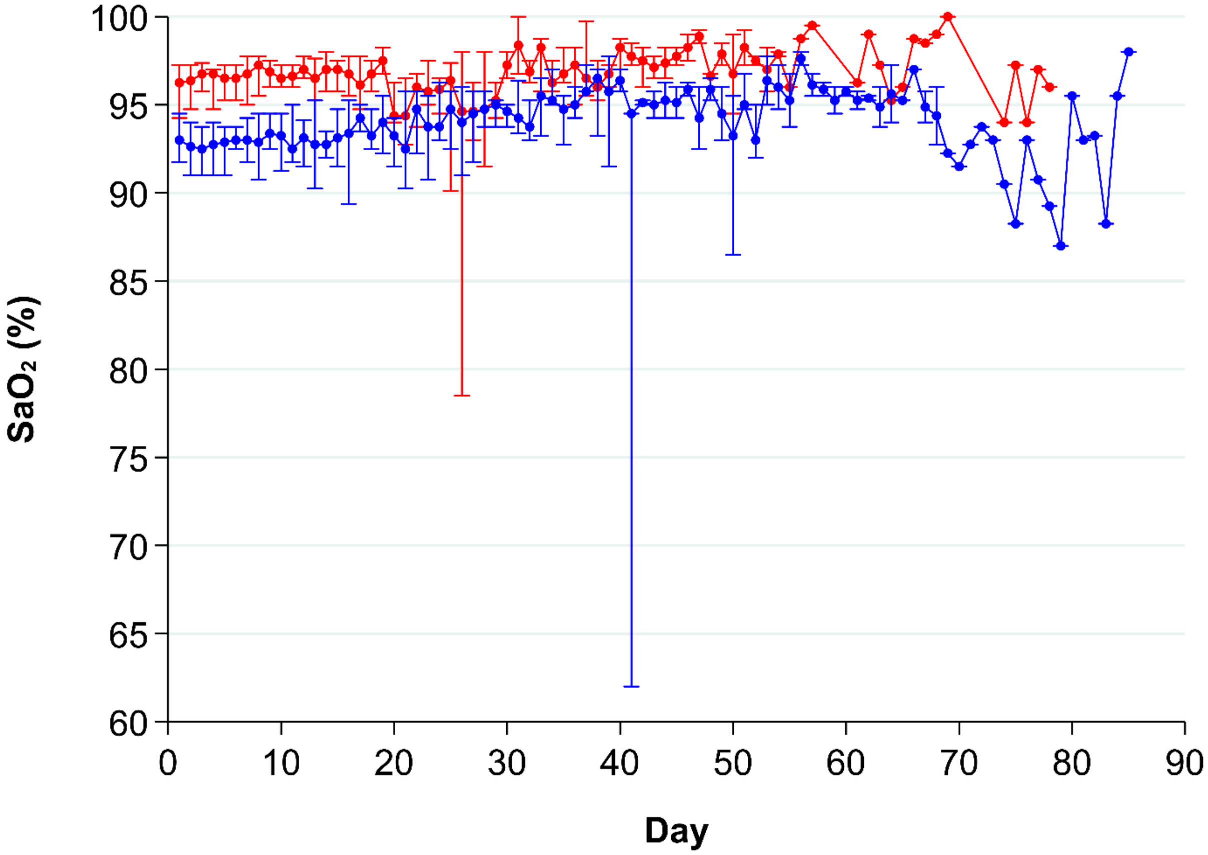

Fig. S3 Daily patient-mean SaO_2

Median values of daily patient-means of arterial oxygen saturation (SaO_2) stratified according to oxygenation target allocation for the 90-day intervention period in patients with active haematological malignancy.

Daily patient-means were calculated from the registered values corresponding to the 12-hour highest and lowest partial pressure of arterial oxygen. Bars represent interquartile ranges (IQR). Data for SaO_2 were not available for four patients in the lower-oxygenation group and for five patients in the higher-oxygenation group because this parameter was not available in blood gas analyses at one site.
